# Real-time Artificial Intelligence Navigation-Assisted Anatomical Recognition in Laparoscopic Colorectal Surgery

**DOI:** 10.1007/s11605-023-05819-1

**Published:** 2023-08-31

**Authors:** Shunjin Ryu, Keisuke Goto, Takahiro Kitagawa, Takehiro Kobayashi, Junichi Shimada, Ryusuke Ito, Yukio Nakabayashi

**Affiliations:** Department of Digestive Surgery, Kawaguchi Municipal Medical Center, 180, Nishiaraijuku, Kawaguchi City, Saitama 333-0833 Japan

**Keywords:** Artificial intelligence, Navigation, Colorectal surgery, Colorectal cancer, Eureka

## Background

The artificial intelligence (AI) model that Kumazu reported has been improved and can highlight connective tissues and nerves in the surgical field intraoperatively on a monitor.^1^ This improved surgical AI prototype was named Eureka (Anaut, Inc., Tokyo, Japan) and could only be used for research and education now.

We examined whether AI navigation (AIN) using Eureka assists trainees in recognizing nerves during colorectal surgery.

## Methods

**Fig. 1:**
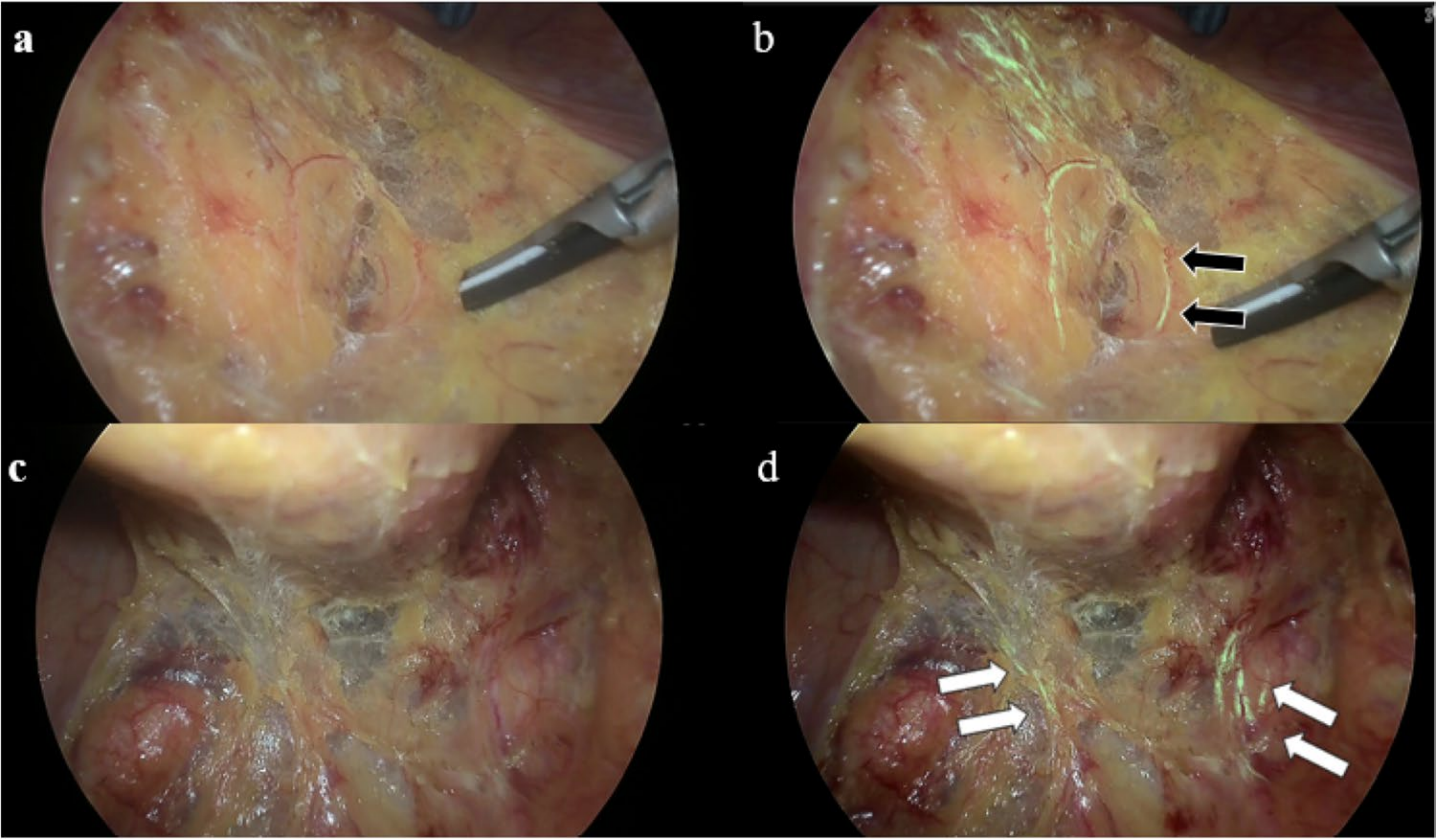
**a** is a typical image of the median approach for sigmoidectomy. **b** is the same scene as **a**, with the AIN. The black arrows indicate the right hypogastric nerve. **c** is a typical image of dissection of the dorsal rectosigmoid. **d** is the same scene as **c**, with the AIN. The white arrows indicate the bilateral hypogastric nerves

**Fig. 2:**
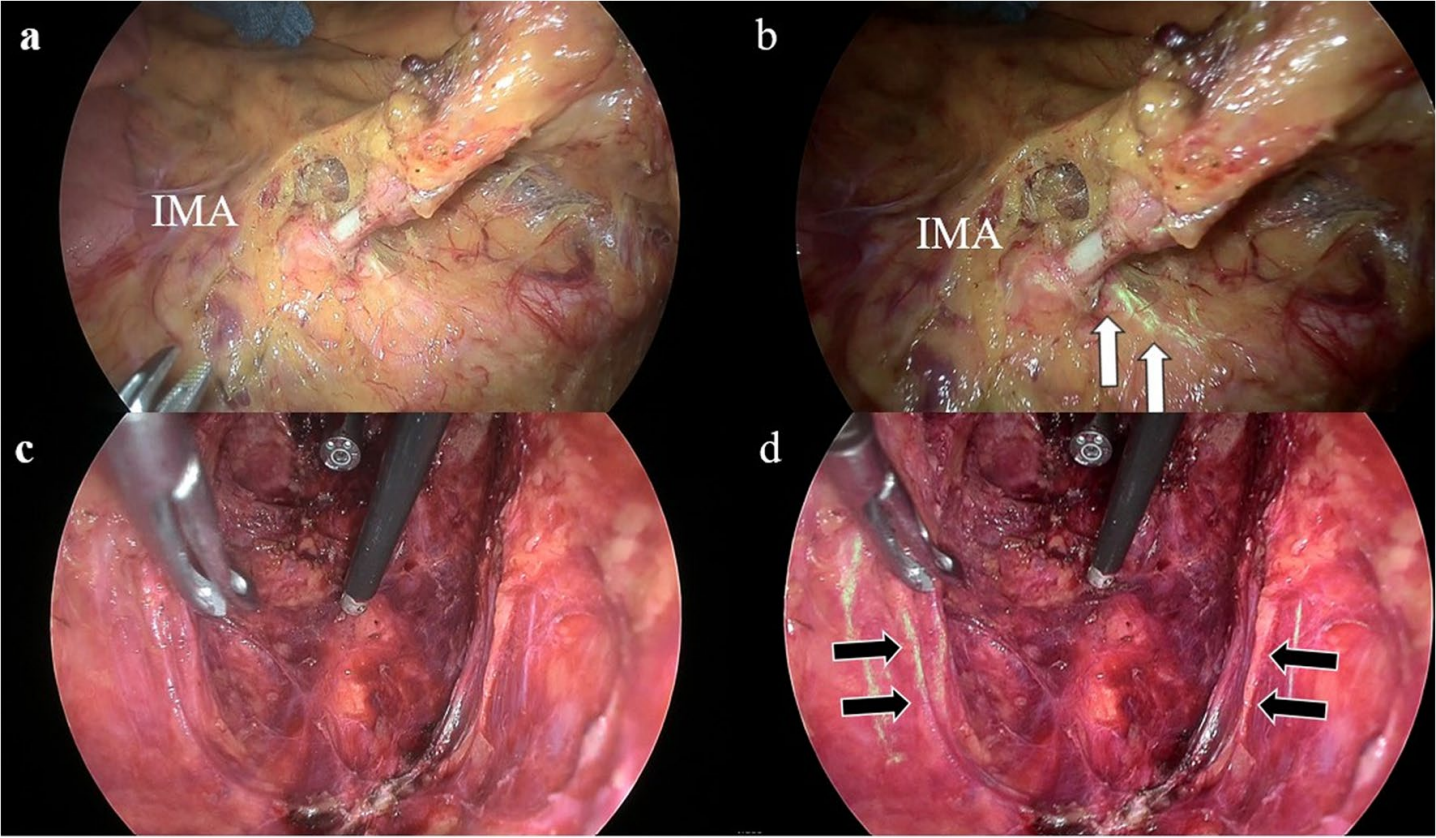
**a** is a typical image of dissection of the dorsal IMA. **b** is the same scene as **a**, with the AIN. The white arrows indicate the lumbar splanchnic nerves. **c** is a typical image after rectal resection. **d** is the same scene as **c**, with the AIN. The black arrows indicate the pelvic visceral nerves (S3, S4)

This study comprised 10 patients who underwent laparoscopic left colorectal surgery between November 2022 and December 2022. All surgeries were performed by one attending doctor who was qualified according to the endoscopic surgical skill qualification system of the Japan Society for Endoscopic Surgery and several trainees.^2^ Whether the attending doctor and trainee were able to recognize each nerve at the same time was examined. If the trainee was unable to recognize nerves, we examined whether viewing AIN monitor connects to the laparoscopic system could assist the trainee in recognizing nerves (Figs. 1 and 2). The AIN monitor was only viewed at the evaluating time. This study was approved by the Research Ethics Committee of our institute (2022–27). All patients provided consent to participate in this study. The results are expressed as the median and interquartile range.

## Results

There were three sigmoid colon cancer patients, six rectal cancer patients, and one sigmoid diverticulum patients. The mean operative duration was 246 [198–272] min, the mean blood loss was 5 [1–9] ml, and the mean duration of postoperative hospital stay was 9 [8–11] days.

The frequencies at which the trainee was not able to recognize the nerves without AIN are as follows: right hypogastric nerve during sigmoid colon mobilization, 9/24 (38%); lumbar splanchnic nerves during dissection of the dorsal inferior mesenteric artery, 8/24 (33%); bilateral hypogastric nerves during dissection of the dorsal rectum, 10/24 (42%); pelvic visceral nerves (S3, S4) during rectal dissection, 12/16 (75%).

When the trainees could not recognize the hypogastric nerves, lumbar splanchnic nerves, AIN aided in anatomical recognition in all cases. However, for the pelvic visceral nerves, AIN provided assistance in 25% of cases.

Differences between 2 junior trainees with less than 5 years of experience and 3 senior trainees with 5–10 years of experience are shown in Table 1.

**Table 1:** Differences in anatomical recognition support with AIN, stratified by years of physician experience

Anatomical structure	Junior trainee	Senior trainee	*P* value
Right hypogastric nerve	7/8(87.5%)	2/16 (12.5%)	0.0007
Lumbar splanchnic nerves	8/8 (100%)	0/16 (0%)	< 0.0001
Bilateral hypogastric nerves	8/8 (100%)	2/16 (12.5%)	< 0.0001
Pelvic visceral nerves	0/5 (0%)	3/11 (27.27%)	0.5089

## Discussion

AIN using Eureka provided intraoperative real-time visualization of nerves, which was safe for education and did not negatively affect surgical outcomes.

Because Eureka can highlight nerves on recorded surgical videos, AIN may improve the efficiency of trainees’ self-study.

Although the utility of AIN varies by anatomical structure, AIN can potentially assist in anatomical recognition and contribute to surgical education for especially young trainee.

## Data Availability

The data that support the findings of this study are available from the corresponding author upon request.
